# Harnessing the small intestinal axis to resolve systemic inflammation

**DOI:** 10.3389/fimmu.2022.1060607

**Published:** 2022-11-15

**Authors:** Mark Bodmer, Andrea Itano, Iain McInnes

**Affiliations:** ^1^ Research and Development, Evelo Biosciences, Cambridge, MA, United States; ^2^ College of Medical, Veterinary and Life Sciences, University of Glasgow, Glasgow, United Kingdom

**Keywords:** immunity, small intestine, mucosa, oral tolerance, T-cell, medicines

## Abstract

This Perspective presents the potential of the Small Intestinal Axis, a sub-division of the Gut-immune Axis, to modulate systemic inflammation based on sensing contents of the gut lumen. Gut mucosal immunity regulates tolerance to food and gut contents and is a significant factor in maintaining systemic homeostasis without compromising immunity to pathogens. This is achieved through anatomical structures and signaling pathways that link the tolerogenic potential of the proximal small intestine to systemic immunity. Non-live preparations of microbes isolated from human small intestinal mucosa, and the extracellular vesicles (EVs) which they shed, can resolve systemic inflammation without systemic exposure after oral delivery. The mechanism involves primary interactions with pattern recognition receptors followed by trafficking of immune cells through mesenteric lymph nodes. This generates in the periphery a population of circulating CD4^+^ T cells which have regulatory function but an atypical FoxP3^-^ phenotype. There is no modification of the resident gut microbiome. Discoveries using this novel approach of targeting mucosal microbial elements to the tolerogenic proximal regions of the small intestine are revealing some of the mysteries of the relationship between the gut and immune system.

## Introduction

Since ancient times the intestines have been considered integral to human well-being. The ancient Egyptians removed intestines before mummification and placed them in canopic jars protected by the falcon-headed God, Qebehsenuef, one of the four sons of Horus (1, 2). In modern times, we possess a growing scientific understanding of a profound influence of the gut on the function of the immune system. The large mucosal surface of the small intestine, in particular, functions as an immune sensory organ which relays information and instructions throughout the body based on molecular sensing of luminal content, including food and microbes (3).

Inferences of the gut’s influence on immune homeostasis and pathology have focused on the association of changes in composition of gut microbiota measured in stool. This represents the microbial content of the colon and is distinct from that of the small intestine. The colonic microbiota are widely considered as a vital organ for health, despite the observation that individuals who have a total colectomy are generally healthy. This Perspective presents an alternative view centered on a sub-division of the gut-immune axis, the small intestinal axis (“Sintax”), that links small intestinal mucosal immunity to systemic immunity. We describe the therapeutic potential of the small intestinal axis based on an understanding of gut mucosal immunity and of harnessing its links to the periphery.

Recent studies exploring oral agents that modulate peripheral inflammation *via* the small intestinal axis (4, 5) have provided unexpected insights that bridge the fields of oral tolerance, mucosal immunity, and the gut microbiome. This raises the possibility of medicines which induce resolution of systemic inflammation using mechanisms similar to those essential to prevent inflammatory responses to the high burden of daily exposure to food antigens in the proximal small intestine (6).

Pabst and Mowat (7) noted that, “There is an important difference between tolerance to gut bacteria and tolerance to food proteins: whereas tolerance to food protein induced *via* the small intestine affects local and systemic immune responses, tolerance to gut bacteria in the colon does not attenuate systemic responses.” We are seeking to bridge this gap with the systemic effects of mucosa-resident organisms on mechanisms of oral tolerance in the proximal small intestine.

## On the evolutionary origins of the gut-immune axis

Theodosius Dobzhansky stated that, “Nothing in biology makes sense except in the light of evolution” (8).

The last common ancestor (LCA) of all animals is thought to have been a worm-like creature, possibly *Ikaria wariootia*, found in the Ediacaran of South Australia and estimated to be ~550 million years old (9). The possibility that a worm is the LCA is consistent with predictions based on phylogenetic analysis of modern animals such as *Platynereis dumerilii* (10).

The significance for the gut-immune axis arises from the striking anatomical similarity between worm and small intestine, which is an evolutionary descendent of the LCA. During embryogenesis the proto-gut forms in the first few cell divisions and all other tissues of the body arise from gut epithelial cells. The worm/gut can be regarded as the core ancestral element of the organism, deriving the nutrients from the environment required to drive the energetics of life (11). This evolutionary core must control the myriad tissues and organs that have evolved to enable diverse animals to adapt to widely varied habitats. The control systems are the anatomical immune, neural and metabolic networks which radiate from the gut throughout the body.

With this evolutionary perspective it becomes intuitive to understand that the small intestine can exert physiological control of bodily function. Indeed, it must, as it is a central force of life within all animals. This insight informs our understanding of the small intestinal axis and the creation of new types of medicines.

## The conundrum of the gut microbiome

The gut microbiome has been characterized as an essential organ for good health (12). Enormous amounts of data have been generated on the content and diversity of the gut microbiota (13, 14). Associations of gut microbial variation with disease have been reported across most areas of medicine (15, 16). Given ease of access, the majority of data on gut microbiome composition is from stool samples. The colon has a high microbial abundance, several orders of magnitude greater than the upper parts of the intestine (17, 18).

There is, however, compelling evidence that the colonic microbiome may not be generally essential to health. This is most evident in adult patients who have had a total colectomy. Case-control longitudinal follow-up studies after surgery show no adverse effects on health related to the loss of the colon and its microbiota (19). Two large cohort studies may suggest even a reduced risk of cardiovascular disease (20) and type 2 diabetes (21) after colectomy. This is inconsistent with the assumption that a colonic microbiome is essential to good health but remains compatible with the notion that dysbiosis can lead to adverse effects on health reflecting the overgrowth of organisms with which the host has not evolved to co-exist.

Small intestine resection is less commonly performed. However, observations of patients with concomitant inflammatory diseases following Roux-en-Y surgery for obesity suggest that alterations in function of the small intestine can result in inflammation resolution in peripheral tissues (22). Although the mechanism of this effect is unknown, it suggests that the proximal small intestine can influence systemic inflammation.

Evidence that modulating gut mucosal immunity can lead to tolerance and reduced systemic inflammation is long-standing (23). Two related ideas prompted us to think in a different way about the gut-immune axis and how to modulate it therapeutically:

Firstly, there are multiple microbiomes within the GI tract. The colonic and small intestinal mucosal microbiome are distinct, with a density about six orders of magnitude lower in the duodenum and three orders lower in the distal ileum compared to the colon (24). Microbes resident in the small intestine mucosa are more likely to influence the small intestinal axis than those in stool (25) due to proximity to host cells in the gut epithelium and their evolutionary requirement to adapt to survive in the host environment.

Secondly, it may be possible to select individual microbes or microbial elements which trigger tolerogenic sensory systems in the proximal small intestine for control of systemic inflammation using similar mechanisms to those of food tolerance (6, 26).

We propose a new avenue for the discovery of orally delivered medicines which explores the intersection between mechanisms of oral tolerance and small intestinal mucosal microbiota. This hypothesis led to identification of agents which harness tolerogenic gut-immune networks resulting in broad spectrum systemic inflammation resolution arising from direct interaction with host cells in the small intestine, unrelated to content or change in the microbiota.

## The role of small intestinal mucosal immunity

The intestinal mucosal immune system must permit safe nutrient absorption and offer protection from pathogens. Much of the protective function arises from physical, chemical and biochemical barriers in the GI tract (27), ensuring that pathogens never enter the body. A primary requirement of gut mucosal immunity is prevention of adverse inflammatory effects of food consumption (7) and exposure to innocuous microbes whilst still allowing immunity to pathogens.

We have probed the systemic impact of the small intestinal axis using EDP1867 and EDP1815, two preparations of single strains of small intestine mucosa-derived bacteria and the extracellular vesicles which they naturally shed. These can be orally delivered with inflammation resolving effects which match the efficacy of a wide range of comparator anti-inflammatory drugs in animal models.

Ramani et al. in 2022 (4) published the first report of the pharmacology and mechanisms of EDP1867 on the small intestinal axis. EDP1867 is a preparation of a monoclonal strain of the bacterium, *Veillonella parvula*, isolated from the ileum of a healthy human donor who had a total colectomy 25 years earlier. The preparation comprised a mixture of bacterial cells and their extracellular vesicles (EVs). It was γ-irradiated to ensure that the mechanism of action was due to direct action on host cells in the gut rather than GI colonization or modification of the resident microbiota. EDP1867 was therapeutically effective after oral administration in a range of animal models covering Th1, Th2 and Th17 inflammation.

This systemic effect of oral tolerance with a small intestine mucosa-derived microbial preparation has also been shown with EDP1815, prepared from a strain of *Prevotella histicola* isolated from a duodenal biopsy of a human donor (28). EDP1815 is an obligate anaerobe which is killed by exposure to oxygen following anaerobic fermentation. It has shown efficacy in a variety of inflammatory models affecting a range of organs and tissues (29–31). Both EDP1867 and EDP1815 were shown to be gut-restricted after oral administration, demonstrating that the effects in the periphery were due to the transmission of an immunological signal that originated in the gut rather than to systemic absorption of the drug.

## Immunogeography of the intestines

The distribution of immune cells, epithelial cells and gene expression throughout the gut is highly non-uniform (32, 33) suggesting differential functions along its length. There is anatomical segregation of the types of dendritic cells (DCs) that migrate to mesenteric lymph nodes draining the small intestine and the colon (34). The mechanisms that mediate oral tolerance, linking the gut to control of systemic inflammation, appear to be prevalent in the proximal small intestine where food is first encountered in the intestines.

Prevention of inflammatory responses to this xenobiotic load requires that initial interactions are regulatory, consistent with the distribution of immune cells along the intestine. The duodenum and upper parts of the jejunum contain substantial numbers of CD103^+^CD11b^+^ DCs (32), a class of DC unique to the intestine with a role in the generation of regulatory T cells (35).

Esterhazy et al. (36) used direct injection of antigen into proximal and distal regions of the gut to determine the responses generated in the associated draining lymph nodes (LNs). They reported proximal small intestine-draining LNs preferentially giving rise to tolerogenic responses and the distal LNs to pro-inflammatory T cell responses.

We have unpublished results showing a related phenomenon with EDP1815. In animal experiments on EDP1815 formulation, 1mm tablets small enough to be dosed orally to mice were given polymer coatings that released the contents either in the proximal small intestine or the distal ileum/colon. The results complemented those of Esterhazy et al. (36). Only the proximal release formulation of EDP1815 resulted in a significant systemic anti-inflammatory effect in a T cell driven delayed-type hypersensitivity (DTH) model of inflammation. In contrast to Esterhazy et al. there was no pro-inflammatory effect of distal release.

Orally delivered microbial preparations showed similar efficacy to parenterally administered dexamethasone, suggesting the significant control that the proximal small intestinal can exert on systemic inflammation.

## Mechanisms of inflammation resolution *via* the small intestinal axis

Descriptions of the phenomenon of systemic tolerance after oral administration of antigens date to the early 1900s. In 1911 Wells and Osborne investigated biological reactions to vegetable proteins (23). Whilst their primary interest was in anaphylactic reactions to intraperitoneally administered proteins, they commented that, “Experiments showed that continuous feeding with a vegetable protein rendered guinea-pigs immune to this protein, so that they could not be sensitized to it.” Thus, over 110 years ago it was known that peripheral and gut mucosal immune responses to the same antigen were quite different. Not only did exposure in the gut by feeding not induce anaphylaxis, but it actually prevented sensitization, similar to an observation about LPS noted below (37).

Nearly 100 years later Parameswaran et al. (38) reported that oral tolerance to OVA also elicited a protective effector response to a recombinant OVA-expressing strain of *Salmonella enterica*. The mechanism is not understood but suggests that there are intestinal mucosa immune mechanisms which can simultaneously elicit systemic tolerance and effector responses depending on the context in which the antigen is seen by the host.

The host recognition contacts with foreign matter are pattern recognition receptors (PRRs), first postulated by Janeway in 1989 (39). We now know that there are dozens of PRRs which integrate signals from a huge array of ligands to modulate innate immunity. These ligand-receptor interactions are conserved across domains of life (40). In many parts of the body they function as “danger” signals alerting the immune system to threat (41). However, gut mucosal immune wiring is not the same as in the periphery. Receptors which generate proinflammatory responses in the periphery can have opposite anti-inflammatory or regulatory effects when activated in the gut. Examples are the actions of Toll-like receptors TLR4 and TLR5 (42, 43). Agents such as lipopolysaccharide (LPS) that are inflammatory when given intravenously can be anti-inflammatory after oral administration (44). Indeed, repeated oral administration of LPS can be protective against subsequent intravenous challenge (37), reminiscent of original observations of Wells and Osborne in 1911 (23).

**Figure 1:**
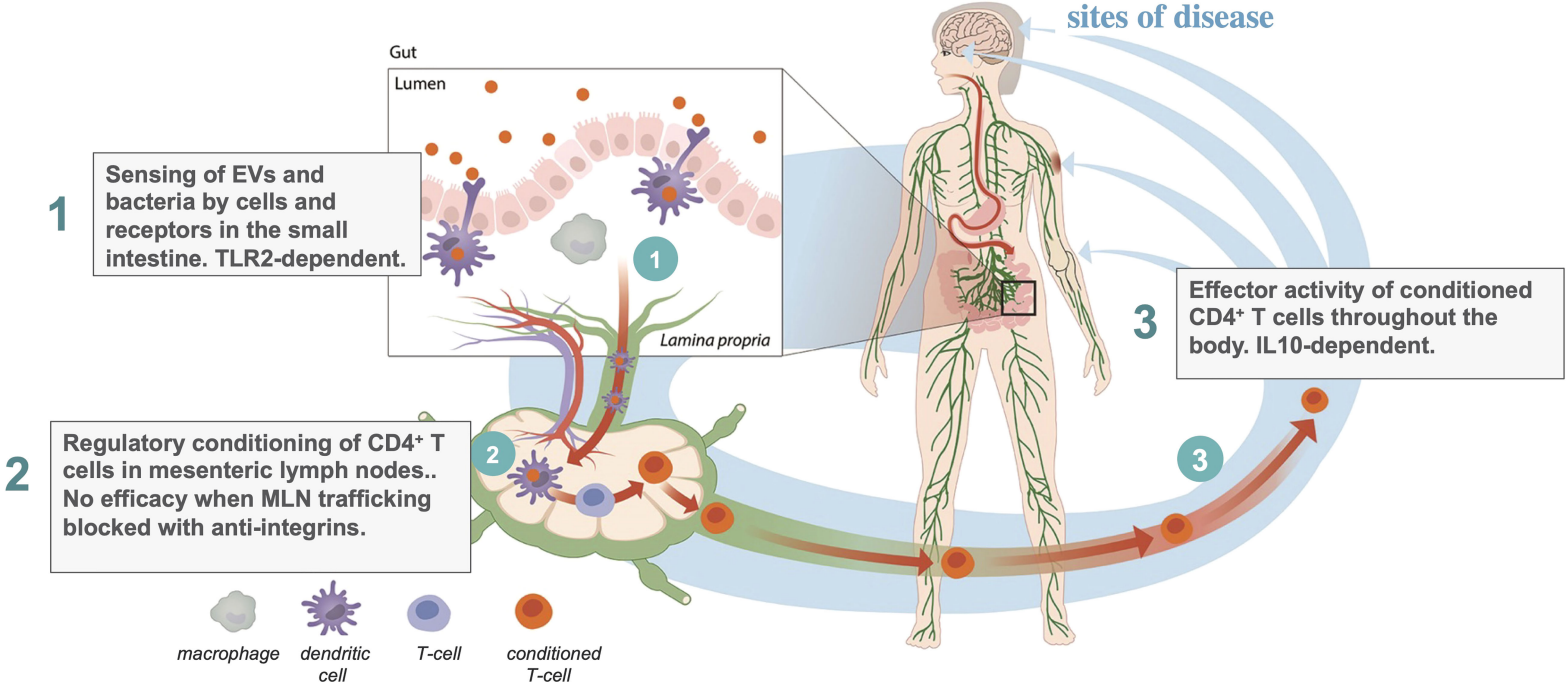
Three step model of inflammation resolution in the small intestinal axis.

The action of EDP1867 and EDP1815 in the small intestinal axis is dependent on transfer of immune signals from the intestine to the periphery. The mechanism of this effect has been traced through three steps (**Figure 1**): first, primary interactions with pattern recognition receptors; second, trafficking through mesenteric lymph nodes, resulting in; third, the generation in the periphery of circulating CD4^+^ T-cells whose ability to regulate inflammation can been shown by adoptive cell transfer into untreated animals. The study of EDP1867 mentioned above (4), experimentally elucidated this mechanistic chain of events.

TLR2 engagement in the gut was required to initiate a cascade of events leading to the generation of a population of CD4^+^ cells which have inflammation resolving effects in the periphery. TLR2 is expressed on many immune cell types and has a promiscuous range of ligands and functions (45). As well as stimulating inflammation it has been reported to induce dendritic cell mediated tolerance under some conditions (46) and even to maintain gut barrier function enhancing immune homeostasis (47). In our studies with orally-delivered EDP1867, antibody-mediated blockade of TLR2 prevented the *in vivo* efficacy in a mouse model of inflammation. Although we have not yet characterized the primary target cell in the small intestine, this demonstrates that TLR2 is at least one of the molecular targets required to mediate systemic effects, and that the relevant cells bearing TLR2 are likely to be more abundant in the proximal intestine. Subsequent unpublished studies comparing the pharmacology of a range of microbial strains, extracellular vesicles (EVs), and an orally delivered TLR2 agonist have revealed that TLR2 engagement is necessary but not sufficient for the systemic anti-inflammatory effect.

Mesenteric lymph nodes (MLN) are the critical interface between gut mucosal and peripheral immunity. If lymphocyte trafficking through MLNs is blocked using a cocktail of anti-integrin antibodies against α4β7 (LPAM−1) and CD62L, then there is no signal transfer from the gut to the periphery, and no efficacy of EDP1867. The anti-integrins do not, themselves, block the inflammatory response. This provides key evidence that immunological signals in the gut have a route to the periphery. This requirement for MLN trafficking to regulate peripheral inflammation by signals in the gut is an interesting inverse to the efficacy of vedolizumab in the treatment of inflammatory bowel diseases (48, 49). Vedolizumab is an antibody that prevents lymphocyte trafficking to the gut by blocking human α4β7, leading to a reduction in bowel inflammation, the inverse effect to our observations of signalling from the gut to the periphery.

We next determined the peripheral cellular effect of the primary events in the gut and MLNs. Consistent with earlier reports on tolerance induced by gliadin and PSA (26, 50), treatment with EDP1867 led to the generation of a population of CD4^+^ T cells that could adoptively transfer the inflammation resolving effect from donor animals treated with EDP1867 to naïve recipient animals which had a subsequent challenge. This is also the case for animals treated with EDP1815 (manuscript in preparation). Whilst the generation of therapeutically effective T cells in donors was TLR2-dependent, their anti-inflammatory effector function in recipients was not. The converse was the case for IL-10, which was not required in the donors but was for effector function in recipients. This T cell dependent mechanism results in long-lasting effects weeks after the cessation of treatment. This was a breakthrough finding, both in understanding the immune mechanism resulting in the peripheral anti-inflammatory effects, and for the therapeutic potential of this approach. By engaging oral tolerance mechanisms, the small intestinal axis can generate circulating T cells with regulatory function which induce generalized inflammation resolution without any apparent adverse effects in either preclinical or clinical studies to date.

Since the publication on EDP1867 we have observed that the anti-inflammatory T cells induced *via* the small intestinal axis are noncanonical FoxP3^-^ CD4 cells. Although differing from most reports of Tregs, this fits with a growing literature on FoxP3-independent T cells that nevertheless have regulatory functions. Van der Veeken et al. (5) reported a peripheral Treg transcriptional program which is induced by events in the intestine and is independent of FoxP3. Hong et al. (26) described populations of noncanonical helper T cells which appear to mediate non-responsiveness to oral gliadin. Sefik et al. (51) observed a distinct population of RORγ^+^ T-cells induced by intestinal symbionts. Johnson et al. (50, 52) reported that capsular polysaccharide A from *B. fragilis* induced T cells capable of regulating airway hyperreactivity that are not canonical FoxP3^+^ Tregs. This latter report is particularly interesting because it showed that a non antigen-specific systemic anti-inflammatory agent acting in the gut could regulate the OVA antigen specificity used to generate airway inflammation.

## What mediates microbial function within the small intestinal axis

Microbial and eukaryotic cells shed lipid nanoparticles which comprise part of the cell membrane and cytoplasmic molecular contents, variously known as extracellular vesicles, outer membrane particles, or exosomes. These may be the major mediators of inter-cellular communication (53–55). Unlike the cells themselves, they are small enough for Brownian motion (56) which enables them to diffuse across the local microenvironment to signal to neighboring cells and tissues.

Most preparations of microbes contain EVs. Being sub-microscopic they are not generally tracked. The earliest descriptions of bacterial EVs by electron microscopy were in the 1960s (57, 58). It is now emerging that the EVs may mediate biological effects which have been ascribed to microbial cells. Thus oral delivery of EVs of *Akkermansia muciniphila* was protective in a mouse model of DSS colitis (59) and also able to improve gut permeability with possible benefits in a range of metabolic diseases (60), activities that have been attributed previously to the microbe itself.

EDP1867 contains EVs. When the EVs were separated from the microbial cells and independently tested in an *in vivo* model of DTH, the purified EVs exhibited the full anti-inflammatory effects that were observed in the microbial preparation containing both cells and EVs. All of our mechanistic observations on the signaling path from gut to periphery are common to EV and microbial preparations. Although roughly a thousand-fold smaller in volume than the parent microbial cells, we have been unable to detect them outside of the gut in our studies after oral administration suggesting that their primary site of action is the intestinal mucosa. In a direct comparison, EVs resolved systemic inflammation after oral but not intravenous or intraperitoneal administration.

Taken together, we have observed systemic anti-inflammatory effects that depend on primary interactions in the small intestine with both non-viable microbial cells and EV preparations that cannot colonize the gut microbiome. Since EVs are not living entities, they provide the clearest evidence for a drug-type rather than a microbiological-type mechanism, likely using the potent mechanisms required for normal food tolerance. The pharmacology cannot be attributed to a probiotic type of action.

A point to note is that the functional activities of bacteria and EVs in host interactions are often attached to taxonomic names, from species up to phylum level. There is not a sufficiently meaningful definition of a bacterial species (61) to allow function to be clearly associated with taxonomy. The variability within what is classed as a species is sufficient for the biology of interactions with the immune system to differ even between strains of the same species. Bacterial function in the gut-immune axis must be defined at the level of the individual clonal strain as much as by taxonomy.

## Clinical potential of targeting the small intestinal axis

The broad-spectrum and potent inflammation resolving activity described above in animal models, supported by the CD4 T cellular mechanism of action, has the potential to translate to humans to address the unmet clinical needs of people with immune-mediated inflammatory diseases (IMID). Despite significant recent therapeutic advances, immune homeostasis is not restored in a large majority of IMID patients nor is remission achieved sufficiently often.

Our observations, interpreted in light of the many contributions cited in this article, suggest a path to solving Pabst and Mowat’s dilemma of the differential immunological effects of the upper and lower gut by delivering components of microbes to the proximal tolerogenic regions at pharmacological doses. This is supported by a weight of preclinical data, and now clinical trial results which are in preparation for publication. EDP1815 has to date been administered to over 700 people, including patients with psoriasis, atopic dermatitis or COVID-19. Encouraging efficacy has shown that this principle of addressing gut tolerance mechanisms with a mucosal microbial preparation can have effects in humans. A phase 2 study produced clinically meaningful outcomes amongst 250 patients with mild and moderate psoriasis (62). Another phase 2 study in 400 patients with atopic dermatitis is under way. There have been no safety or tolerability issues in studies to date that differ from placebo controls. These agents can be manufactured at large scale and reasonable cost, which establishes the potential of a new class of effective, safe and affordable oral anti-inflammatory medicines.

## Concluding remarks

We have described effector functions of killed mucosal microbes and their EV constituents acting on the small intestinal axis to resolve systemic inflammation without systemic exposure. Gut mucosal immunity not only regulates tolerance to food and gut contents, but it is also a significant factor in maintaining systemic non-inflamed homeostasis without compromising parenteral immunity to pathogens. As well as contributing to the understanding of the gut-immune axis, our observations suggest the possibility of a major advance in the treatment of inflammatory diseases.

## Data availability statement

The raw data supporting the conclusions of this article will be made available by the authors, without undue reservation.

## Author contributions

The first draft of the manuscript was written by MB and revised by AI and IM. All authors contributed to the article and approved the submitted version.

## Acknowledgments

We thank Dr. Duncan McHale, Dr. Douglas Maslin and Dr. Simba Gill for helpful suggestions and discussions. We thank Dr. Noubar Afeyan for insights into the physics of extracellular vesicles. And finally, we thank members of Evelo Biosciences for all their work to generate results that are mentioned in the article.

## Conflict of interest

MB and AI are employees of Evelo Biosciences Inc. IM is a non-executive member of the Board of Directors of the company.

Authors MB and AI declare that this study received funding from Evelo Biosciences Inc. The funder was involved in the study design, collection, analysis, interpretation of data, and the decision to submit it for publication.

## Publisher’s note

All claims expressed in this article are solely those of the authors and do not necessarily represent those of their affiliated organizations, or those of the publisher, the editors and the reviewers. Any product that may be evaluated in this article, or claim that may be made by its manufacturer, is not guaranteed or endorsed by the publisher.

## References

[1] Nunn JF . Ancient Egyptian medicine. Trans Med Soc Lond (1996) 113:57–68. 10326089

[2] Walker-Smith J . A god for guts. Gut. (2002) 50(6):886–7. doi: 10.1136/gut.50.6.886 PMC1773223 12010895

[3] Furness JB Rivera LR Cho HJ Bravo DM Callaghan B . The gut as a sensory organ. Nat Rev Gastroenterol Hepatol (2013) 10(12):729–40. doi: 10.1038/nrgastro.2013.180 24061204

[4] Ramani K Cormack T Cartwright ANR Alami A Parameswaran P Abdou M . Regulation of peripheral inflammation by a non-viable, non-colonizing strain of commensal bacteria. Front Immunol (2022) 13:768076. doi: 10.3389/fimmu.2022.768076 35185874 PMC8847375

[5] van der Veeken J Campbell C Pritykin Y Schizas M Verter J Hu W . Genetic tracing reveals transcription factor Foxp3-dependent and Foxp3-independent functionality of peripherally induced treg cells. Immunity (2022) 55(7):1173–84.e7. doi: 10.1016/j.immuni.2022.05.010 35700740 PMC9885886

[6] Pinheiro-Rosa N Torres L Oliveira MA Andrade-Oliveira MF Guimaraes MAF Coelho MM . Oral tolerance as antigen-specific immunotherapy. Immunother Adv (2021) 1(1):ltab017. doi: 10.1093/immadv/ltab017 35919733 PMC9327124

[7] Pabst O Mowat AM . Oral tolerance to food protein. Mucosal Immunol (2012) 5(3):232–9. doi: 10.1038/mi.2012.4 PMC3328017 22318493

[8] Dobzhansky T . Nothing in biology makes sense except in the light of evolution. Am Biol Teacher (1973) 35(3):125. doi: 10.2307/4444260

[9] Evans SD Hughes IV Gehling JG Droser ML . Discovery of the oldest bilaterian from the ediacaran of south Australia. Proc Natl Acad Sci USA (2020) 117(14):7845–50. doi: 10.1073/pnas.2001045117 PMC7149385 32205432

[10] Christodoulou F Raible F Tomer R Simakov O Trachana K Klaus S . Ancient animal microRNAs and the evolution of tissue identity. Nature (2010) 463(7284):1084–8. doi: 10.1038/nature08744 PMC2981144 20118916

[11] Lane N . The vital question : energy, evolution, and the origins of complex life. In: First American edition. New York: W. W. Norton & Company (2015). p. 360.

[12] O'Hara AM Shanahan F . The gut flora as a forgotten organ. EMBO Rep (2006) 7(7):688–93. doi: 10.1038/sj.embor.7400731 PMC1500832 16819463

[13] Lloyd-Price J Mahurkar A Rahnavard G Crabtree J Orvis J Hall AB . Strains, functions and dynamics in the expanded human microbiome project. Nature. (2017) 550(7674):61–6. doi: 10.1038/nature23889 PMC5831082 28953883

[14] Truong DT Tett A Pasolli E Huttenhower C Segata N . Microbial strain-level population structure and genetic diversity from metagenomes. Genome Res (2017) 27(4):626–38. doi: 10.1101/gr.216242.116 PMC5378180 28167665

[15] Chaudhari SN McCurry MD Devlin AS . Chains of evidence from correlations to causal molecules in microbiome-linked diseases. Nat Chem Biol (2021) 17(10):1046–56. doi: 10.1038/s41589-021-00861-z PMC8480537 34552222

[16] Vujkovic-Cvijin I Sklar J Jiang L Natarajan L Knight R Belkaid Y . Host variables confound gut microbiota studies of human disease. Nature. (2020) 587(7834):448–54. doi: 10.1038/s41586-020-2881-9 PMC7677204 33149306

[17] de Vos WM Tilg H Van Hul M Cani PD . Gut microbiome and health: mechanistic insights. Gut (2022) 71(5):1020–32. doi: 10.1136/gutjnl-2021-326789 PMC8995832 35105664

[18] Donaldson GP Lee SM Mazmanian SK . Gut biogeography of the bacterial microbiota. Nat Rev Microbiol (2016) 14(1):20–32. doi: 10.1038/nrmicro3552 26499895 PMC4837114

[19] Roberts SE Williams JG Yeates D Goldacre MJ . Mortality in patients with and without colectomy admitted to hospital for ulcerative colitis and crohn's disease: record linkage studies. BMJ (2007) 335(7628):1033. doi: 10.1136/bmj.39345.714039.55 17977817 PMC2078633

[20] Wu CC Hsu TW Yeh CC Lee CH Lin MC Chang CM . The impact of colectomy on the risk of cardiovascular disease among patients without colorectal cancer. Sci Rep (2020) 10(1):2925. doi: 10.1038/s41598-020-59640-w 32076006 PMC7031401

[21] Wu CC Lee CH Hsu TW Yeh CC Lin MC Chang CM . Is colectomy associated with the risk of type 2 diabetes in patients without colorectal cancer? a population-based cohort study. J Clin Med (2021) 10(22):5313–23. doi: 10.3390/jcm10225313 PMC8622203 34830601

[22] Sparks JA Halperin F Karlson JC Karlson EW Bermas BL . Impact of bariatric surgery on patients with rheumatoid arthritis. Arthritis Care Res (Hoboken) (2015) 67(12):1619–26. doi: 10.1002/acr.22629 PMC4662646 26018243

[23] Wells HG Osborne TB . The biological reactions of the vegetable proteins i. anaphylaxis. J Infect Diseases. (1911) 8(1):66–124. doi: 10.1093/infdis/8.1.66

[24] Kastl AJ Jr. Terry NA Wu GD Albenberg LG . The structure and function of the human small intestinal microbiota: Current understanding and future directions. Cell Mol Gastroenterol Hepatol (2020) 9(1):33–45. doi: 10.1016/j.jcmgh.2019.07.006 31344510 PMC6881639

[25] Moran C Sheehan D Shanahan F . The small bowel microbiota. Curr Opin Gastroenterol (2015) 31(2):130–6. doi: 10.1097/MOG.0000000000000157 25603402

[26] Hong SW Krueger PD Osum KC Dileepan T Herman A Mueller DL . Immune tolerance of food is mediated by layers of CD4(+) T cell dysfunction. Nature (2022) 607(7920):762–8. doi: 10.1038/s41586-022-04916-6 PMC10336534 35794484

[27] Yang I Nell S Suerbaum S . Survival in hostile territory: the microbiota of the stomach. FEMS Microbiol Rev (2013) 37(5):736–61. doi: 10.1111/1574-6976.12027 23790154

[28] Itano A Cormack T Ramani K Hilliard-Barth KL Ponichtera HE Ganguly T . 16003 orally administered EDP1815, a monoclonal strain of prevotella histicola, has potent systemic anti-inflammatory effects. J Am Acad Dermatol (2020) 83(6):AB52. doi: 10.1016/j.jaad.2020.06.297

[29] Marietta EV Murray JA Luckey DH Jeraldo PR Lamba A Patel R . Suppression of inflammatory arthritis by human gut-derived prevotella histicola in humanized mice. Arthritis Rheumatol (2016) 68(12):2878–88. doi: 10.1002/art.39785 PMC5125894 27337150

[30] Mangalam A Shahi SK Luckey D Karau M Marietta E Luo N . Human gut-derived commensal bacteria suppress CNS inflammatory and demyelinating disease. Cell Rep (2017) 20(6):1269–77. doi: 10.1016/j.celrep.2017.07.031 PMC5763484 28793252

[31] Marietta E Horwath I Meyer S Khaleghi-Rostamkolaei S Norman E Luckey D . Administration of human derived upper gut commensal prevotella histicola delays the onset of type 1 diabetes in NOD mice. BMC Microbiol (2022) 22(1):8. doi: 10.1186/s12866-021-02406-9 34983374 PMC8729070

[32] Mowat AM Agace WW . Regional specialization within the intestinal immune system. Nat Rev Immunol (2014) 14(10):667–85. doi: 10.1038/nri3738 25234148

[33] Elmentaite R Kumasaka N Roberts K Fleming A Dann E King HW . Cells of the human intestinal tract mapped across space and time. Nature. (2021) 597(7875):250–5. doi: 10.1038/s41586-021-03852-1 PMC8426186 34497389

[34] Houston SA Cerovic V Thomson C Brewer J Mowat AM Milling S . The lymph nodes draining the small intestine and colon are anatomically separate and immunologically distinct. Mucosal Immunol (2016) 9(2):468–78. doi: 10.1038/mi.2015.77 26329428

[35] Bain CC Montgomery J Scott CL Kel JM Girard-Madoux MJH Martens L . TGFbetaR signalling controls CD103(+)CD11b(+) dendritic cell development in the intestine. Nat Commun (2017) 8(1):620. doi: 10.1038/s41467-017-00658-6 28931816 PMC5607002

[36] Esterhazy D Canesso MCC Mesin L Muller PA de Castro TBR Lockhart A . Compartmentalized gut lymph node drainage dictates adaptive immune responses. Nature (2019) 569(7754):126–30. doi: 10.1038/s41586-019-1125-3 PMC6587593 30988509

[37] Holst H Edqvist LE Kindahl H . Reduced response to intravenous endotoxin injections following repeated oral administration of endotoxin in the pig. Acta Veterinaria Scandinavica (1993) 34(4):405–19. doi: 10.1186/BF03548185 PMC8112536 8147294

[38] Parameswaran N Samuvel DJ Kumar R Thatai S Bal V Rath S . Oral tolerance in T cells is accompanied by induction of effector function in lymphoid organs after systemic immunization. Infect Immun (2004) 72(7):3803–11. doi: 10.1128/IAI.72.7.3803-3811.2004 PMC427424 15213121

[39] Janeway CA Jr . Approaching the asymptote? evolution and revolution in immunology. Cold Spring Harb Symp Quant Biol (1989) 54 Pt 1:1–13. doi: 10.1101/sqb.1989.054.01.003 2700931

[40] Li D Wu M . Pattern recognition receptors in health and diseases. Signal Transduct Target Ther (2021) 6(1):291. doi: 10.1038/s41392-021-00687-0 34344870 PMC8333067

[41] Matzinger P . Tolerance, danger, and the extended family. Annu Rev Immunol (1994) 12:991–1045. doi: 10.1146/annurev.iy.12.040194.005015 8011301

[42] Chassaing B Ley RE Gewirtz AT . Intestinal epithelial cell toll-like receptor 5 regulates the intestinal microbiota to prevent low-grade inflammation and metabolic syndrome in mice. Gastroenterology (2014) 147(6):1363–77.e17. doi: 10.1053/j.gastro.2014.08.033 25172014 PMC4253564

[43] Lu P Sodhi CP Yamaguchi Y Jia H Prindle T Jr. Fulton WB . Intestinal epithelial toll-like receptor 4 prevents metabolic syndrome by regulating interactions between microbes and intestinal epithelial cells in mice. Mucosal Immunol (2018) 11(3):727–40. doi: 10.1038/mi.2017.114 PMC6131112 29363671

[44] Inagawa H Kohchi C GS . Oral administration of lipopolysaccharides for the prevention of various diseases: Benefit and usefulness. Anticancer Res (2011) 31(7):2431–6. 21873155

[45] Oliveira-Nascimento L Massari P Wetzler LM . The role of TLR2 in infection and immunity. Front Immunol (2012) 3:79. doi: 10.3389/fimmu.2012.00079 22566960 PMC3342043

[46] Geisel J Kahl F Muller M Wagner H Kirschning CJ Autenrieth IB . IL-6 and maturation govern TLR2 and TLR4 induced TLR agonist tolerance and cross-tolerance in dendritic cells. J Immunol (2007) 179(9):5811–8. doi: 10.4049/jimmunol.179.9.5811 17947654

[47] Cario E . Barrier-protective function of intestinal epithelial toll-like receptor 2. Mucosal Immunol (2008) 1 Suppl 1:S62–6. doi: 10.1038/mi.2008.47 19079234

[48] Feagan BG Rutgeerts P Sands BE Hanauer S Colombel JF Sandborn WJ . Vedolizumab as induction and maintenance therapy for ulcerative colitis. N Engl J Med (2013) 369(8):699–710. doi: 10.1056/NEJMoa1215734 23964932

[49] Sandborn WJ Feagan BG Rutgeerts P Hanauer S Colombel JF Sands BE . Vedolizumab as induction and maintenance therapy for crohn's disease. N Engl J Med (2013) 369(8):711–21. doi: 10.1056/NEJMoa1215739 23964933

[50] Johnson JL Jones MB Cobb BA . Bacterial capsular polysaccharide prevents the onset of asthma through T-cell activation. Glycobiology (2015) 25(4):368–75. doi: 10.1093/glycob/cwu117 PMC4339875 25347992

[51] Sefik E Geva-Zatorsky N Oh S Konnikova L Zemmour D McGuire AM . MUCOSAL IMMUNOLOGY. individual intestinal symbionts induce a distinct population of RORgamma(+) regulatory T cells. Science (2015) 349(6251):993–7. doi: 10.1126/science.aaa9420 PMC4700932 26272906

[52] Johnson JL Jones MB Cobb BA . Polysaccharide-experienced effector T cells induce IL-10 in FoxP3+ regulatory T cells to prevent pulmonary inflammation. Glycobiology (2018) 28(1):50–8. doi: 10.1093/glycob/cwx093 PMC5972631 29087497

[53] Nahui Palomino RA Vanpouille C Costantini PE Margolis L . Microbiota-host communications: Bacterial extracellular vesicles as a common language. PLoS Pathog (2021) 17(5):e1009508. doi: 10.1371/journal.ppat.1009508 33984071 PMC8118305

[54] Raposo G Stahl PD . Extracellular vesicles: a new communication paradigm? Nat Rev Mol Cell Biol (2019) 20(9):509–10. doi: 10.1038/s41580-019-0158-7 31324871

[55] Diaz-Garrido N Badia J Baldoma L . Microbiota-derived extracellular vesicles in interkingdom communication in the gut. J Extracell Vesicles (2021) 10(13):e12161. doi: 10.1002/jev2.12161 34738337 PMC8568775

[56] Newburgh R Peidle J Rueckner W . Einstein, Perrin, and the reality of atoms: 1905 revisited. Am J Phys (2006) 74(6):478. doi: 10.1119/1.2188962

[57] Knox KW Vesk M Work E . Relation between excreted lipopolysaccharide complexes and surface structures of a lysine-limited culture of escherichia coli. J Bacteriol (1966) 92(4):1206–17. doi: 10.1128/jb.92.4.1206-1217.1966 PMC276396 4959044

[58] Chatterjee SN Das J . Electron microscopic observations on the excretion of cell-wall material by vibrio cholerae. J Gen Microbiol (1967) 49(1):1–11. doi: 10.1099/00221287-49-1-1 4168882

[59] Kang CS Ban M Choi EJ Moon HG Jeon JS Kim DK . Extracellular vesicles derived from gut microbiota, especially akkermansia muciniphila, protect the progression of dextran sulfate sodium-induced colitis. PLoS One (2013) 8(10):e76520. doi: 10.1371/journal.pone.0076520 24204633 PMC3811976

[60] Chelakkot C Choi Y Kim DK Park HT Ghim J Kwon Y . Akkermansia muciniphila-derived extracellular vesicles influence gut permeability through the regulation of tight junctions. Exp Mol Med (2018) 50(2):e450. doi: 10.1038/emm.2017.282 29472701 PMC5903829

[61] Caro-Quintero A Konstantinidis KT . Bacterial species may exist, metagenomics reveal. Environ Microbiol (2012) 14(2):347–55. doi: 10.1111/j.1462-2920.2011.02668.x 22151572

[62] Maslin D . (2022). A phase 2 study investigating the effect of EDP1815, an orally-delivered, anti-inflammatory, gut-restricted commensal microbe in the treatment of mild and moderate plaque psoriasis, in: 2022 AAD nnual Meeting, Boston, MA, 26 March 2022.

